# Is Psychological Distress Associated with Self-Perceived Health, Perceived Social Support and Physical Activity Level in Spanish Adults with Diabetes?

**DOI:** 10.3390/jpm13050739

**Published:** 2023-04-27

**Authors:** Angel Denche-Zamorano, Jofre Pisà-Canyelles, Sabina Barrios-Fernandez, Raquel Pastor-Cisneros, José C. Adsuar, Miguel Angel Garcia-Gordillo, Damián Pereira-Payo, María Mendoza-Muñoz

**Affiliations:** 1 Promoting a Healthy Society Research Group (PHeSO), Faculty of Sport Sciences, University of Extremadura, 10003 Cáceres, Spain; denchezamorano@unex.es (A.D.-Z.);; 2 Health, Economy, Motricity and Education Research Group (HEME), Faculty of Sport Sciences, University of Extremadura, 10003 Cáceres, Spain; 3 Ability Research Group, Occupation, Participation, Sustainability and Quality of Life, Nursing and Occupational Therapy College, University of Extremadura, 10003 Cáceres, Spain; 4 Universidad Autónoma de Chile, Talca 3467987, Chile; 5 Research Group on Physical and Health Literacy and Health-Related Quality of Life (PHYQOL), Faculty of Sport Sciences, University of Extremadura, 10003 Caceres, Spain; 6 Departamento de Desporto e Saúde, Escola de Saúde e Desenvolvimento Humano, Universidade de Évora, 7004-516 Évora, Portugal

**Keywords:** psychological well-being, health behaviour, exercise, social support, diabetes

## Abstract

Depressive and anxiety symptoms are common in people with type 1 and type 2 diabetes mellitus. Physical activity and social support may prevent or reduce psychological distress in this population. This study aimed to analyse the associations between psychological distress, self-perceived health (SPH), perceived social support (PSS) and physical activity level (PAL) in adults with a diabetes mellitus diagnosis from Spain. A cross-sectional study based on data from the Spanish National Health Survey (ENSE2017) with a final sample of 1006 individuals with diabetes mellitus aged between 15 and 70 years, who completed the Adult Questionnaire. Some of the items from this survey were taken from existing questionnaires: the Goldberg General Health Questionnaire (GHQ-12) for Mental Health status and psychological distress, the Duke-UNC-11 Functional Social Support Questionnaire for PSS and the International Physical Activity Questionnaire (IPAQ) for PAL. A descriptive analysis and non-parametric statistical tests were carried out, including correlation analysis, multiple binary logistic regression and linear regression model calculations. It was found that SPH was related to PAL (*p* < 0.001): positive SPH prevalence was higher in the Active and Very Active groups (*p* < 0.05). Weak inverse correlations were found between the GHQ-12 and the PAL (rho: −0.230; *p* < 0.001) and PSS (rho: −0.234; *p* < 0.001). Physical inactivity and lower PSS were risk factors for negative SPH and worst physiological outcomes. Thus, higher PAL and PSS were positively correlated with SPH and lower psychological stress in Spanish adults with diabetes mellitus.

## 1. Introduction

Diabetes is a major health burden with an increasing global prevalence [1]. It is recognised as a leading chronic disease causing disability and a main cause of mortality [2]. Currently, almost 500 million people worldwide have a diabetes diagnosis, with estimations to increase to 578 million by 2030 and 700 million by 2045 [3]. Moreover, diabetes is related to multiple physical and psychological diseases, including cardiovascular, kidney diseases, ulcers and depression, among others [4].

People with diabetes present an increased risk of mental disorders, including depression or anxiety when compared with the overall population [5,6,7,8]. Diabetes and mental disorders comorbidities lead to worse quality of life [7,9], as they result in poorer glycaemic control, greater disability and unhealthier behaviours [7,9,10,11,12]. Hence, mental health status screening and assessments are needed to avoid negative effects on the patient’s increased social burden, participation restrictions for attending social events or performing physical activity (PA), thus aggravating physical and mental health [9,13]. Self-perceived health (SPH) is an individual’s perception of their health status, representing a widely used indicator for its reliability and simplicity, since it provides relevant information [14,15]. Moreover, negative SPH is associated with higher morbidity and death from a variety of causes of relative risks when compared to more positive SPH [16]. Another important factor is perceived social support (PSS), defined as the individual’s subjective perception of their environmental support [17]. Thus, poorer PSS is associated with worse health and increased anxiety and depression symptoms [18]. Thus, family members and close friends play an important role in the care of people with diabetes [19].

As some individuals with diabetes experience a poor quality of life, tools to manage their symptomatology are needed. Therefore, PA is recommended, as it improves mental and physical health in the general population, although these studies are scarce in people with diabetes [20]. Associations between PA and the lack of sleep, social problems or depressive symptoms have been reported [4,21]. For this reason, engagement in PA has an important role in preventing acute and chronic complications [13]. Furthermore, PA can help in glycaemia management, delaying other cardiometabolic risk factors and contributing to an improved quality of life and psychological well-being [13,22,23]. Moreover, PA must be provided by PA professionals, who can assess and implement PA programs according to an individual’s characteristics and preferences [13,22,23,24,25]. Hence, active lifestyle behaviours, including PA performance (aerobic physical exercise and strength and muscle strengthening [26]) and 150 min of moderate or 75 min of vigorous PA per week [25], as recommended for the general population by the World Health Organization).

With the above in mind, this study aimed to explore the associations between psychological distress with self-perceived health (SPH), perceived social support (PSS) and physical activity level (PAL) in adults with diabetes in Spain in the period before the COVID-19 pandemic, analysing sex differences.

## 2. Materials and Methods

### 2.1. Design, Data Sources and Ethical Concerns

A descriptive cross-sectional correlational study, based on data extracted from the Spanish National Health Survey 2017 (ENSE2017) [27] was performed. The ENSE2017 provides health data and indicators of Spanish residents aged 15 years and older before the COVID-19 pandemic. The ENSE2017 uses individual surveys carried out by trained and accredited staff from the Spanish Ministry of Health, Consumer Affairs and Social Welfare and the Spanish National Institute of Statistics. The surveys were carried out between October 2016 and October 2017 and datasets are available at https://www.sanidad.gob.es/estadisticas/microdatos.do (accessed on day 16 June 2022).

As these data were anonymized, they are considered to be non-confidential, so no approval from an ethics committee was necessary to work with them. Nevertheless, before the interviews, all the participants were properly informed about all aspects involved in the study and signed informed consent was obtained.

### 2.2. Participants

The ENSE2017 total sample size included 23,089 participants of 15 years and over who were residents in Spain. A three-stage stratified sampling system was used: census sections, family households in those sections and individuals in those households [27].

Eligibility criteria included being aged between 15 and 70 years; having a diabetes diagnosis (item Q.25a.12), which includes patients from any of the diabetes mellitus groups of metabolic diseases (including type 1 and type 2 diabetes mellitus); and having answered all the items on psychological distress (Q.47.1–Q.47.12) and PA (Q.113–Q.117) in the ENSE2017 Adult Questionnaire [28].

Then, 5312 participants were excluded for age (over 70 years); 16,759 for not having Diabetes; and 12 for answering “do not know/no answer” to the Psychological Distress (10 participants) or PA (2 participants) items, resulting in a final sample of 1006 participants. Only for analyses that included a PSS variable were participants who responded “do not know/no answer” to items Q.130.1–Q.130.11 (those referring to the Duke-UNC-11 Questionnaire) excluded (52 individuals).

### 2.3. Procedures

Data were extracted from the following variables collected through the Adult Questionnaire [28] in the ENSE2017: sex (female or male), age (years), height (cm) and weight (kg), in addition to the answers to the items Q.21, Q.25a.12, Q.47.1–Q.47.12, Q.113–Q.117 and Q.130.1–Q.130.11. The following variables were derived from the above items:

Body mass index (BMI): from weight and height (in kg/m^2^).

Diabetes status: from answers to item Q.25a.12 (“Have you ever had diabetes?” Options: Yes, No, Do not know or No answer (NS/NC)).

Self-Perceived Health (SPH): from answers to item Q.21 (“In the last twelve months, would you say that your health has been very good, good, fair, poor, very poor?”): “good” and “very good” answers were considered as positive SPH, while “fair”, “poor” and “very poor” were considered as negative SPH.

Mental Health: derived from answers to items Q.47.1–Q.47.12, belonging to the Goldberg General Health Questionnaire (GHQ-12), a multidimensional scale that assesses psychological distress and mental health. In this questionnaire, questions can take values between 0 and 3 (total range within 0 to 36 points), where 0 represents good mental health and 36 represents the worst [29]. The GHQ-12 is considered a valid and reliable instrument in the Spanish context [30], which allows the following mental health dimension assessments [31,32]:

- Successful Coping: derived from the total of the items Q.47.1, Q.47.3, Q.47.4, Q47.7, Q47.8 and Q.47.12, with punctuation ranging from 0 to 18, where 0 is the best and 18 is the worst coping level, and has an external validity of 0.82 with a *p*-value of 0.001.
- Self-esteem: derived from the sum of the items Q.47.6, Q.47.9, Q.47.10 and Q.47.11, ranging from 0 to 12, where 0 is the best and 12 is the worst, and has an external validity of 0.70 with a *p*-value of 0.001.
- Stress: derived from the sum of the items Q.47.2, Q.47.5 and Q.47.9; ranging from 0 to 9, where 0 is the best and 9 is the worst, and has an external validity of 0.75, with a *p*-value of 0.001.

Perceived Social Support (PSS): from the items Q.130.1–Q.130.11 corresponding to the Duke-UNC-11 Functional Social Support Questionnaire [33], a tool which analyses participants’ PSS. The answers to the items can take a value between 0 and 5, with a total score between 0 and 55, with 0 being the lowest PSS (poor PSS) and 55 being the highest PSS (a perception of having a large amount of social support). In Spain, less than 32 points are considered to be a low PSS. Moreover, this tool is considered to have excellent reliability and it is considered valid and reliable in the Spanish population [32,34].

Physical activity index (PAI): constructed to quantify the PA performed by the participants. This variable was derived from items Q.113–Q.116, corresponding to the International Physical Activity Questionnaire (IPAQ) Spanish Short version [35]. This PAI formula, adapted from Ness and colleagues, has been already used and described in other publications [36]. This index can take values between 0 and 67.5, with 0 being the lowest PA and 67.5 the highest [37].

Physical activity level (PAL): created by grouping participants according to their PAI scores. However, participants with PAI = 0 were divided into groups termed Inactives and Walkers, according to the answers delivered to subitem Q.117 (“time spent walking in the last 7 days)”. The following values were considered: Inactives (persons with PAI = 0 and answers “No day more than 10 min at a time” in Q.117), Walkers (persons with PAI = 0 and reported walking more than 10 min at a time, at least one day a week), Actives (PAI between 1 and 30) and Very actives (PAI > 30) [32,38].

### 2.4. Statistical Analysis

The Kolmogorov–Smirnov test was used to test data distribution. A descriptive analysis was performed, presenting the continuous variables (age, height, weight, BMI, mental health, successful coping, self-esteem, stress and PSS) through median and interquartile ranges, mean and standard deviations and analysing differences between sexes using the Mann–Whitney U test. Categorical variables (SPH and PAL) were presented by relative and absolute frequencies, analysing possible associations between sex through the chi-square test and differences between sex proportions through a z-test for independent proportions. The same tests were conducted in order to analyse associations between SPH and PAL (chi-square test), and differences between SPH proportions in PAL groups (z-test). Possible baseline differences between mental health variables medians (successful coping, self-esteem and stress) in the different PAL groups were analysed using the Kruskal–Wallis test in the total population and when dividing by sex. Spearman’s correlation coefficients were calculated between the GHQ-12 items and the variables derived from them, and Bonferroni’s correction was used for PAL and PSS, interpreted according to Barrera’s proposal [39]. Multiple binary logistic models were used to study the predictor variables’ effects (age, sex, BMI, PSS and PAL) on SPH. A stepwise linear regression analysis to predict the GHQ-12 scores by mental health, successful coping, self-esteem and stress was performed considering sex, age, BMI, PSS and PAL as independent variables. Cronbach’s alpha reliability index was used to assess the reliability of the scales used: mental health, successful coping, self-esteem, stress and PSS. A significance level lower than 0.001 was required to introduce a new variable into each prediction model. The overall predictive power was evaluated by adjusted R^2^.

## 3. Results

There was insufficient statistical evidence to conclude the assumption of normality in the variables of interest (*p* < 0.001); therefore, non-parametric tests were used. Table 1 provides a descriptive analysis of the sample together with a sex comparison analysis. The median age was 61 years with no significant differences between the sexes. Although the PAL medians were 0 in the total population and the two sexes, significant variances were found between men and women, a trend also reflected in the mean (6.8 vs. 4.7). Nevertheless, no significant dependency relationships were found between PAL and sex. Only 21.7% of the population performed moderate and/or intense PA (24.3% in men and 18.2% in women). Significant differences were found in the medians obtained from men and women in the GHQ-12, both in mental health and in three of its dimensions. No sex differences were found according to the PSS.

A dependency association was found between the PAL and SPH. Thus, Table 2 shows the negative and positive SPH prevalence according to the PAL, displaying differences in the proportions of negative and positive SPH between the Inactives/Walkers and the Active/Very active PAL groups.

**Figure 1:**
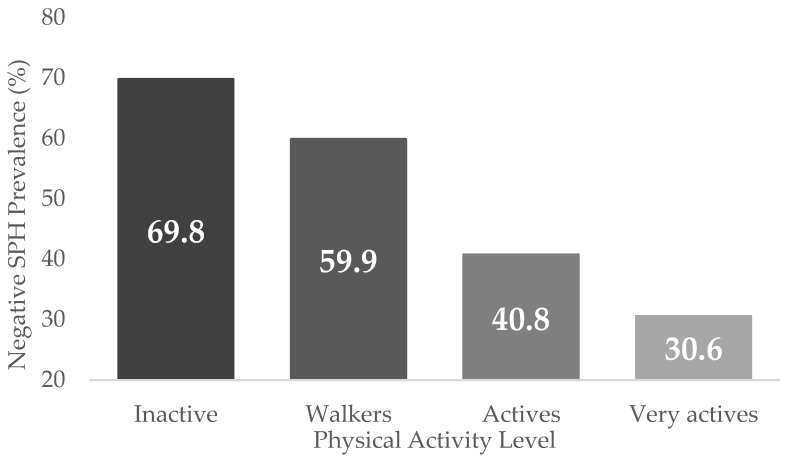
Negative self-perceived health prevalence by physical activity level, according to the ENSE2017.

Figure 1 shows negative SPH prevalence according to the PAL with a difference in proportions close to 40 points between Inactive and Very Active people with diabetes.

The Inactive group scored higher on the GHQ-12. This was also found in the overall population, where the Inactives had a five-point difference in median scores compared to the Very Active group (12 vs. 7). In women, differences between medians reached six points. In the baseline, GHQ-12 medians found significant differences, both in mental health and in all its dimensions (except stress), taking PAL as a factor (Table 3).

The correlations between the PAL and the GHQ-12 scores were inverse, weak to very weak: mental health (rho: −0.220. *p* < 0.001), successful coping (rho: −0.193. *p* < 0.001), self-stem (rho: −0.215. *p* < 0.001) and stress (rho: −0.160. *p* < 0.001) (Table 4). Similarly, weak to very weak inverse correlations were found between the PAL and the GHQ-12 items (*p* < 0.001). Lower PAL was related to greater psychological distress, higher stress and lower scores in the successful coping and self-esteem factors.

Table 5 presents the PSS evaluated using the Duke-UNC-11. Weak inverse correlations were found for mental health scores (*p* < 0.001) and their dimensions: successful coping (r: −0.189. *p* < 0.001), self-confidence (r: −0.230. *p* < 0.001) and stress (r: −0.230. *p* < 0.001). The correlations with each item were weak or very weak.

According to the SPH results, individuals with diabetes who are inactive, female, older and have lower PSS and higher BMI have increased risks of negatively perceiving their health. This model explained 12.2% (Nagelkerke R^2^) of the SPH variance (Table 6).

The linear regression models revealed determination coefficients as shown:

- R^2^ = 17.8%, positively explained by mental health (constant: β = 21.22, t = 19.30, *p* < 0.001; PSS: β = −0.204, t = −10.35, *p* < 0.001; PAL: β = −1.38, t = −6.33, *p* < 0.001; sex: β = 1.82, t = 5.54, *p* < 0.001)
- R^2^ = 15.5%, positively explained by successful coping (constant: β = 10.24, t = 23.93, *p* < 0.001; PSS: β = −0.075, t = −9.73, *p* < 0.001; PAL: β = −0.472, t = −5.57, *p* < 0.001; sex: β = 0.61, t = 4.81, *p* < 0.001)
- R^2^ = 14.7%, positively explained by self-esteem (constant: β = 7.23, t = 13.51, *p* < 0.001; PSS: β = −0.647, t = −9.25, *p* < 0.001; PAL: β = −0.647, t = −6.11, *p* < 0.001; sex: β = 0.679, t = 4.25, *p* < 0.001)
- R^2^ = 13.0%, positively explained by stress (constant: β = 5.86, t = 13.12, *p* < 0.001; PSS: β = −0.068, t = −8.48, *p* < 0.001; PAL: β = −0.408, t = −4.62, *p* < 0.001; sex: β = 0.751, t = 5.63, *p* < 0.001)

The reliability coefficients (Cronbach’s alpha) for each scale used were: mental health (0.91), successful coping (0.87), self-esteem (0.87), stress (0.82) and perceived social support (0.92).

## 4. Discussion

This study aimed to analyse the associations between psychological distress with self-perceived health (SPH), perceived social support (PSS) and physical activity level (PAL) in Spanish adults with diabetes. Based on our results, a dependent relationship was found between SPH and PAL. Those who were Inactive had a higher negative SPH prevalence, while the Very Active individuals showed a higher positive SPH prevalence. According to the GHQ-12 scores, inactive individuals presented more psychological distress than the rest of the groups with scores above 12, considered the cut-off point for some forms of psychological distress [40]. In all the mental health dimensions, scores were higher (which indicate a worse mental health state) in the inactive population compared to the rest of the population. Inverse relationships were discovered between GHQ-12 and PAL scores and PSS. Higher PAL and higher PSS were linked to lower scores on psychological distress agreed to the GHQ-12.

International PA guidelines suggest various recommendations on exercise level for the general population. The Canadian, the USA and Swedish National Guidelines establish a goal of moderate PA of 150 min per week. The Australian Guideline recommends performing PA nearly every day, emphasizing maintenance and age-appropriate exercise. All of these include specific recommendations to reduce sedentary time [25]. Recommendations for people with diabetes do not differ greatly from those for the general population: a total of 150 min of moderate aerobic exercise (50–70% of maximum heart rate) or 75 min of vigorous exercise or a combination of both, 3 days per week, with no more than 2 continuous days omitting exercise [24]. Studies in different regions find that PA adherence may become lower with increasing age. In Spain, only 21.7% of the population perform moderate and/or intense PA (24.3% in men and 18.2% in women), i.e., only two people out of ten perform moderate or intense PA, compared to four out of ten in the general population (46.2% in men and 34.6% in women) [41]. In the UK, 53% of men aged 16–24 years meet PA recommendations, in contrast to 8% in men over 75 years. In women aged 15–54 years, the PAL remains relatively stable with 29–31% meeting the recommendations but then drops to 4% of those over 75 years meeting the standards [25]. People with diabetes have difficulties in meeting these recommendations for reasons related to time constraints (more frequently manifested in women) [42,43] and spending more time in sedentary activities [44].

Regarding psychological functioning, our findings are consistent with previous studies [1,9,45,46]. We found significant differences in the median scores of both men and women on the GHQ-12 in both the total score and its three dimensions. Spanish people with diabetes have worse SPH and psychological well-being than the general Spanish population of the same age and sex without the disease [45], being more likely to suffer from poor mental health and psychological distress [46]. For example, in the UK, the prevalence of mental health problems was 21.6% for people with diabetes compared to 16.3% without diabetes [25]. In another study using the GHQ-12 questionnaire, it was revealed that the mental health of people with prediabetes and diabetes was significantly worse than that of the general population, with more negative mental health attributes related to poor sleep quality or worrying or feeling unhappy or depressed [47].

Dependency relationships were also found between PAL and SPH, such that lower PSS was associated with low engagement in PA. This could be related to lower body image or social anxiety related to the PAL, according to previous work [13]. Significant differences were also found in the GHQ-12 score medians for mental health, taking PAL as a factor. Individuals with diabetes were more likely to have depression, which is reflected by their lack of motivation to change, decreased confidence towards achievement, frustration tolerance and increased concerns about their weight and physical appearance [48]. Thus, depression, fear, frustration tolerance and social isolation must be considered in this population [49,50]. Lower PAL was associated with greater psychological distress, stress and lower self-esteem, all of which are related to lower SPH levels. Likewise, SPH exhibited weak inverse correlations with the PSS, with these factors being a risk factor that creates barriers to exercise. In terms of sex differences, inactive women with low PSS levels, higher BMI and advanced age had a higher risk of poorer mental health across all dimensions. Thus, women with diabetes have a higher mean in the inactivity scores, supporting the notion that, despite demonstrated benefits, physical inactivity indicators and their consequences remain alarming in women [51]. Furthermore, other studies have shown that women have more depressive symptoms than men [52].

The practical applications of this study include a better understanding of the associations between the analysed variables, which include physical, emotional and social ones. Thus, PA can be used as a health promotion tool, so stakeholders should consider not only exercise at the physical or physiological level but also at the psychological and social, regardless of the diabetes type. In the case of people with diabetes, these factors could interfere with the establishment and maintenance of healthy habits, which are key for good glycaemic control and the prevention of the symptomatology and comorbidities of the group of metabolic diseases included in diabetes. Thus, more research about these factors is needed.

This study has several limitations. On the one hand, as this is a cross-sectional study, it is impossible to analyse cause-effect relations but only to identify associations. On the other, due to the ENSE2017 structure, it was not possible to compare how moderate and intense PA is related to mental health, e.g., regarding the type of PA performed, or to discriminate between different types of diabetes, among other things. Moreover, it was also not possible to identify the type of diabetes of every subject, as well as other important health and socio-demographic information (social class, education level, marital status, etc.), as participants were not questioned about this in the ENSE2017. The strength of the relationship and correlations were low, although statistically significant. A larger sample size reveals findings to be close to the overall population reality and demonstrates, compared to smaller samples, that the findings are statistically significant even with weak relationships. Future studies should complement data collection with questionnaires and subjective perception by monitoring objective physiological data, such as body composition, calorie intake and expenditure and PA intensity and duration related to subjective perception for follow-up [53].

## 5. Conclusions

Higher PAL and PSS were positively associated with SPH and lower psychological distress (successful coping, self-esteem and stress) in Spanish adults with diabetes. Physical inactivity and lower PSS were found to be risk factors for negative SPH and worse physiological outcomes.

Although these results need to be confirmed with longitudinal studies, policies and programs to improve PAL and PSS should be considered.

## Figures and Tables

**Table 1:** Descriptive analysis of the sample.

Variables	Total *n* = 1006	Men *n* = 572	Women *n* = 434	p/p *
**Age (Years)**				*p*
Median (IQR)	61 (12)	61 (10)	61 (13)	0.323
Mean (SD)	58.4 (9.2)	58.8 (8.5)	57.9 (10.0)	
**PAI**				*p*
Median (IQR)	0 (0)	0 (0)	0 (0)	0.014
Mean (SD)	5.9 (13.2)	6.8 (14.1)	4.7 (11.7)	
**Mental Health**				*p*
Median (IQR)	11 (6)	10 (5)	11 (7)	<0.001
Mean (SD)	11.4 (5.5)	10.7 (5.0)	12.4 (6.0)	
**Successful Coping**				*p*
Median (IQR)	6 (1)	6 (0)	6 (1)	<0.001
Mean (SD)	6.7 (2.2)	6.4 (1.9)	7.0 (2.4)	
**Self-Esteem**				*p*
Median (IQR)	2 (4)	2 (4)	3 (4)	<0.001
Mean (SD)	2.7 (2.6)	2.4 (2.5)	3.1 (2.8)	
**Stress**				*p*
Median (IQR)	3 (3)	3 (3)	3 (3)	<0.001
Mean (SD)	2.9 (2.2)	2.6 (2.0)	3.3 (2.3)	
**Perceived Social Support**	Total *n* = 946	Men *n* = 531	Women *n* = 415	*p*
Median (IQR)	50 (9)	48 (10)	49 (12)	0.918
Mean (SD)	47.6 (7.4)	46.7 (8.2)	46.7 (8.3)	
**Self-Perceived Health**	Total *n* = 1006	Men *n* = 572	Women *n* = 434	p *
Negative *n* (%)	576 (57.3)	297 (51.9)	279 (64.3) *	<0.001
Positive *n* (%)	430 (42.7)	275 (48.1)	155 (35.7) *	
**PAL**				
Inactives *n* (%)	199 (19.8%)	111 (19.4%)	88 (20.3%)	0.098
Walkers *n* (%)	589 (58.5%)	322 (54.7%)	267 (61.5%)	
Actives *n* (%)	169 (16.8%)	105 (18.4%)	64 (14.7%)	
Very actives *n* (%)	49 (4.9%)	34 (5.9%)	15 (3.5%)	

*n*: participants; %: percentage; IQR: interquartile Range; SD: standard deviation; GHQ-12: Goldberg’s Questionnaire; PAL: physical activity level; *p*: Mann–Whitney U-test *p*-value; p *: chi-square test *p*-value; * significant differences between sex ratios were considered at *p* < 0.05 from pairwise z-test.

**Table 2:** Diabetes prevalence according to the physical activity level and self-perceived health.

	Physical Activity Levels											
Variables	Inactive		Walkers		Active		Very Active					
**Self-Perceived Health**	*n* (%)		*n* (%)		*n* (%)		*n* (%)		X^2^	df	*p*	CC
**Negative**	139 a	(69.8)	353 a	(59.9)	69 b	(40.8)	15 b	(30.6)	47.5	3	<0.001	0.212
**Positive**	60 a	(30.2)	236 a	(40.1)	100 b	(59.2)	34 b	(59.2)				

*n*: participants; %: percentage; X^2^: Pearson’s chi-square; df: degrees of freedom; *p*: *p*-value; CC: contingency coefficient. ab: different letters mean significant proportional differences between PAL groups with *p* < 0.05 from pairwise z-test.

**Table 3:** Goldberg General Health Questionnaire (GHQ-12) dimensions-subscales scores using physical activity level (PAL) as a factor in Spanish adults with diabetes, ENSE2017.

Variables	Total *n* = 1006			Men *n* = 572			Women *n* = 434		
**Mental Health**									
PAL	m (sd)	mdn (IQR)	*p*	m (sd)	mdn (IQR)	*p*	m (sd)	mdn (IQR)	
Inactives	13.6 (6.7)	12 (7)	<0.001	12.9 (6.8)	12 (8)	<0.001	14.5 (6.5)	12 (8)	*p* < 0.001
Walkers	11.4 (5.3)	11 (6)		10.4 (4.3)	10 (5)		12.6 (6.1)	11 (7)	
Actives	10.0 (4.1)	9 (5)		9.9 (4.1)	9 (5)		10.2 (4.0)	10 (5)	
Very actives	8.8 (4.1)	7 (5)		9.2 (4.4)	7 (6)		7.8 (3.2)	6 (5)	
**Successful Coping**									
PAL	m (sd)	mdn (IQR)	*p*	m (sd)	mdn (IQR)	*p*	m (sd)	mdn (IQR)	
Inactives	7.5 (2.8)	7 (4)	<0.001	7.3 (2.7)	6 (1)	<0.001	7.8 (2.8)	6 (4)	*p* < 0.001
Walkers	6.7 (3.9)	6 (1)		6.3 (2.4)	6 (0)		7.1 (2.3)	6 (1)	
Actives	6.1 (1.5)	6 (0)		6.1 (1.5)	6 (0)		6.1 (1.5)	6 (1)	
Very actives	5.9 (1.3)	6 (0)		6.1 (1.2)	6 (0)		5.5 (1.5)	6 (0)	
**Self-Esteem**									
PAL	m (sd)	mdn (IQR)	*p*	m (sd)	mdn (IQR)	*p*	m (sd)	mdn (IQR)	
Inactives	3.7 (3.0)	4 (4)	<0.001	3.5 (3.1)	4 (4)	<0.001	3.9 (2.8)	4 (3)	*p* < 0.001
Walkers	2.7 (2.5)	2 (4)		2.3 (2.1)	2 (4)		3.1 (2.8)	3 (4)	
Actives	2.0 (2.3)	1 (4)		2.0 (2.3)	1 (4)		2.2 (2.4)	2 (4)	
Very actives	1.6 (2.2)	1 (3)		1.7 (2.4)	1 (3)		1.1 (1.7)	0 (2)	
**Stress**									
PAL	m (sd)	mdn (IQR)	*p*	m (sd)	mdn (IQR)	*p*	m (sd)	mdn (IQR)	
Inactives	3.5 (2.3)	3 (3)	<0.001	3.1 (2.4)	3 (4)	0.028	3.9 (2.2)	3 (2)	*p* < 0.001
Walkers	2.9 (2.2)	3 (3)		2.5 (1.9)	3 (2)		3.3 (2.2)	3 (3)	
Actives	2.5 (1.8)	3 (3)		2.5 (1.8)	2 (2)		2.7 (1.8)	3 (3)	
Very actives	1.9 (2.0)	1 (3)		2.0 (2.1)	2 (3)		1.5 (1.9)	1 (3)	

m (mean); sd (standard deviation); mdn (median); IQR (Interquartile range); GHQ-12 (Goldberg’s General Health Questionnaire. In scores between 0 and 36, 0 represents the best mental health level; successful coping (scores from 0 to 18; 0 represents the best coping level); self-esteem (scores 0 to 9; 0 represents the best self-esteem level); and stress (scores 0 to 9; 0 represents no stress); PAL (physical activity level); *p* (*p*-value from Kruskal–Wallis’s test).

**Table 4:** Correlations between the physical activity level (PAL) and the Goldberg General Health Questionnaire (GHQ-12) in Spanish adults with diabetes according to the ENSE2017.

Target Variable	Rho	*p*
**Mental Health**	−0.220	<0.001
Successful Coping	−0.193	<0.001
Self-esteem	−0.215	<0.001
Stress	−0.160	<0.001
1. Have you been able to concentrate well on what you were doing?	−0.125	<0.001
2. Have your worries caused you to lose sleep?	−0.104	0.001
3. Did you feel that you were playing a useful role in life?	−0.191	<0.001
4. Did you feel able to make decisions?	−0.137	<0.001
5. Have you felt constantly overwhelmed and under stress?	−0.150	<0.001
6. Have you had the feeling that you cannot overcome your difficulties?	−0.220	<0.001
7. Have you been able to enjoy your normal daily activities?	−0.203	<0.001
8. Have you been able to cope adequately with your problems?	−0.162	<0.001
9. Have you felt unhappy or depressed?	−0.162	<0.001
10. Have you lost confidence in yourself?	−0.160	<0.001
11. Have you thought of yourself as a worthless person?	−0.180	<0.001
12. Do you feel reasonably happy considering all the circumstances?	−0.176	<0.001

GHQ-12: Goldberg’s General Health Questionnaire; PAL: physical activity level: Inactives (PAI = 0; no walking or PA for more than 10 min at a time); Walkers (PAI = 0; report walking for more than 10 min at a time at least one day a week); Actives (PAI = 1–30); Very Actives (PAI = +30); PAI: physical activity index: scores between 0 and 67.5; Rho: Spearman’s correlation coefficients with Bonferroni’s correction factor *p* = 0.003); *p*: *p*-value.

**Table 5:** Correlations between perceived social support (Duke-UNC-11) and the Goldberg General Health Questionnaire (GHQ-12) in Spanish adults with diabetes, according to the ENSE2017.

Target Variable	Correlations	*p*
**Mental Health**	−0.234	<0.001
Successful Coping	−0.189	<0.001
Self-esteem	−0.230	<0.001
Stress	−0.189	<0.001
1. Have you been able to concentrate well on what you were doing?	−0.189	<0.001
2. Have your worries caused you to lose sleep?	−0.179	<0.001
3. Did you feel that you were playing a useful role in life?	−0.151	<0.001
4. Did you feel able to make decisions?	−0.164	<0.001
5. Have you felt constantly overwhelmed and under stress?	−0.193	<0.001
6. Have you had the feeling that you cannot overcome your difficulties?	−0.192	<0.001
7. Have you been able to enjoy your normal daily activities?	−0.188	<0.001
8. Have you been able to cope adequately with your problems?	−0.204	<0.001
9. Have you felt unhappy or depressed?	−0.228	<0.001
10. Have you lost confidence in yourself?	−0.204	<0.001
11. Have you thought of yourself as a worthless person?	−0.150	<0.001
12. Do you feel reasonably happy considering all the circumstances?	−0.193	<0.001

GHQ-12: Goldberg’s General Health Questionnaire. Duke-UNC-11: Functional Social Support Questionnaire. Correlations: Spearman’s correlation coefficients with Bonferroni’s correction factor having *p* = 0.003; *p*: *p*-value.

**Table 6:** Multiple binary logistic regression analysis for negative self-perceived health.

	B	SE	Wald	df	Sig	Exp(B)	95% CI for EXP(B)	
							Lower	Upper
**Inactive**			29.898	3	0.000			
**Walker**	−0.320	0.191	2.793	1	0.095	0.726	0.499	1.057
**Active**	−1.021	0.235	18.921	1	0.000	0.360 *	0.228	0.571
**Very active**	−1.411	0.370	14.569	1	0.000	0.244 *	0.118	0.503
**Sex (male)**	−0.552	0.143	14.980	1	0.000	0.576 *	0.435	0.762
**PSS**	−0.026	0.009	8.521	1	0.004	0.975 *	0.958	0.992
**IMC**	0.049	0.014	11.977	1	0.001	1.050 *	1.021	1.079
**Age**	0.021	0.008	7.733	1	0.005	1.022 *	1.006	1.037
**Constant**	−0.398	0.736	0.292	1	0.589	0.672		

B: understandarised beta; SE: standard error of regression; Wald: Wald’s chi-square test; df: degree of freedom; Sig: statistical significance; Exp(B): odds ratio; CI: confidence interval; * statistically significant at *p* < 0.05.

## Data Availability

Data are available under reasonable request to the corresponding author.
